# The future of hepatology: Preventing liver disease together with the public, scientists and artificial intelligence

**DOI:** 10.1016/j.jhepr.2026.101846

**Published:** 2026-04-22

**Authors:** Benjamin P.M. Laevens, Corinna Meeßen, Frank P. Pijpers, Tony Bruns, Yazhou Chen, Rohit Loomba, Niharika Jakhar, Katrin Arning, Kai M. Schneider, Carolin V. Schneider

**Affiliations:** 1 Department of Medicine III, University Hospital RWTH Aachen, Aachen, Germany; 2 Korteweg–de Vries Institute for Mathematics, University of Amsterdam, Amsterdam, The Netherlands; 3 Division of Gastroenterology and Hepatology, University of California at San Diego, San Diego, USA; 4 Risk Perception and Communication Teaching and Research Unit, RWTH Aachen University, Aachen, Germany; 5 Department of Medicine 1, University Hospital Carl Gustav Carus Dresden, Technische Universitaet (TU), Dresden, Germany

**Keywords:** Participatory medicine, Citizen science, Cross-disciplinary

## Abstract

**Background and Aims:**

The coming decades will bring important challenges for medicine, including hepatology, such as rising healthcare costs and the increasing role of artificial intelligence (AI). Addressing these challenges depends partly on how the scientific community builds public trust and support for long-term policy solutions. Here, we explore how broader societal involvement in hepatology through participatory medicine, centred on collaboration between the general public, clinicians, researchers, and intelligent systems, could contribute to research, prevention, and public engagement. Such approaches stimulate partnerships that benefit both lay contributors and researchers.

**Methods:**

Examples from other scientific domains, including astronomy (*Galaxy Zoo*, involving more than 200,000 volunteers classifying galaxies), molecular biology (*EteRNA*, a gamified platform where tens of thousands of players solve RNA-folding puzzles), meteorology (*WOW*, integrating data from citizen weather stations) and traffic monitoring (*Telraam*, where citizens deploy street-level traffic sensors) show how citizen participation and AI can form co-learning loops. Drawing inspiration from these four case studies, we developed six new participatory hepatology projects.

**Results:**

These projects fall into two categories based on participants. Four are public led: *Liver Zoo* (medical imaging annotation), *Liver Cache* (geocaching-based prevention and awareness), *LiverQuest* (gamified prevention and behavioural reflection) and *Heporama* (community-driven aflatoxin surveillance). Two are patient centric: *Liver Cancer Wisdom Bank* (patient knowledge repository) and *Liver4Mind* (neurocognitive monitoring in cirrhosis and encephalopathy).

**Conclusion:**

These proposals illustrate how participatory approaches can strengthen trust, public engagement, and innovation in hepatology, supporting societal involvement in research and prevention. Intended as catalysts for dialogue, we invite the hepatology community to shape these and related participatory initiatives, thereby strengthening communication and public education while positioning hepatology at the forefront of innovative and trust-building medicine.

**Impact and implications:**

This study sets out the case for expanding participatory medicine in hepatology beyond patient-centred research to also include the general public, addressing persistent gaps in prevention, awareness, and representative data in an era of increasing AI use. By adapting established citizen science models from other disciplines into six hepatology-specific project archetypes, the study shows how public engagement can support earlier risk awareness and prevention while providing complementary behavioural, environmental, and perceptual insights, beyond those available in clinical settings. These findings are particularly relevant for hepatologists, public health researchers, and policymakers searching for scalable ways to engage the general population, including individuals at risk but not yet medically treated. In practice, this work helps existing patient-led initiatives broaden their scope, and helps researchers design new public-facing participatory initiatives where few currently exist.

## Highlights

• Participatory medicine can strengthen trust and public engagement in hepatology
• Citizen science and AI co-learning loops offer models for hepatology research
• Four public-led and two patient-centric hepatology projects are proposed
• Gamified and community tools can support liver disease prevention and awareness

## Graphical abstract

**Figure:**
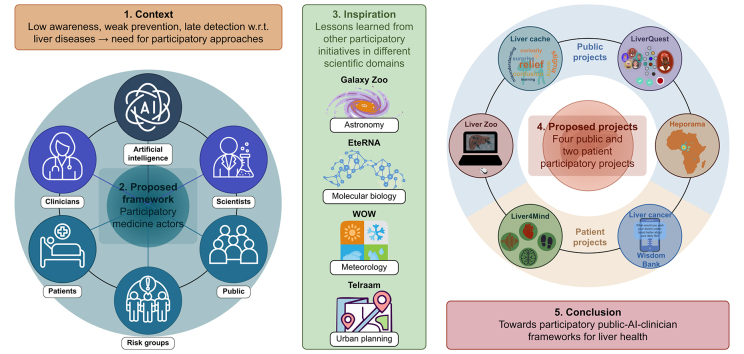

## Introduction

The term ‘citizen science’ (CS) is defined as:

*‘Scientific work undertaken by members of the general public, often in collaboration with or under the direction of professional scientists and scientific institutions’*,^1^

and has been used in fields ranging from biology,^2^ including ornithology^3^ to astrophysics.^4^ It can encompass activities such as data collection, classification tasks, and collaborative hypothesis generation,^2^ allowing large-scale projects to exist that would be unfeasible through research alone.^4^ In its most collaborative form, CS is a bi-directional exchange: a platform for mutual learning, shared decision-making, and co-produced knowledge between scientists and the public,^5^ which can increase public trust.^6^

**Fig. 1:**
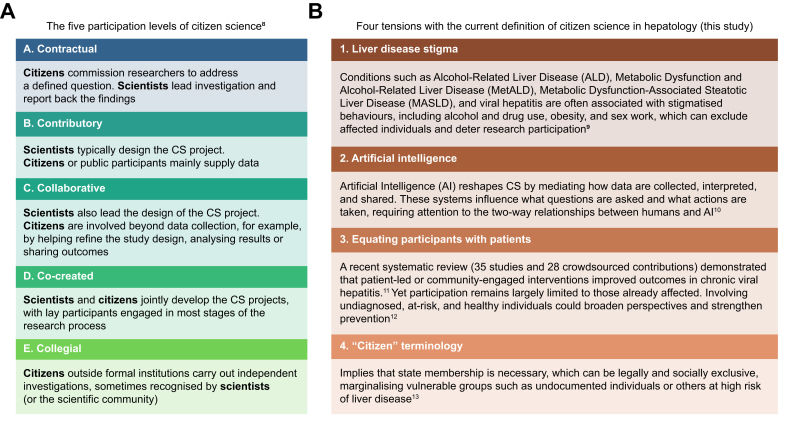
Levels of participation in CS and key tensions in hepatology. Public participation in scientific research can take many forms. (A) A widely used typology^8^ distinguishes five models of CS, based on the depth and structure of engagement, in contrast to (B) the four tensions we identify with the use of this definition in hepatology.[9], [10], [11], [12], [13] CS, citizen science.

Yet in medicine, and hepatology in particular, public involvement remains more limited. To date, CS, in the sense of participation by any member of the public, not only patients, has seen only limited implementation in hepatology. The NAFLD EU Stepchange project is a rare example, where six citizen scientists worked alongside researchers in a 12-week non-alcoholic fatty liver disease (NAFLD) study involving 31 participants, co-shaping data collection and interpretation to test the value and challenges of CS in liver research.^7^ Several factors might contribute to this limited uptake, including the focus of hepatology on basic science and clinical trials, the complexity of liver disease, and the limited structured frameworks or funding for participatory research. Beyond these practical barriers, we also identify four broader tensions that complicate the application of CS in hepatology (Fig. 1B).

### Expanded participatory medicine

To address these tensions, we chose an approach situated within an expanded framing of *‘*participatory medicine’ (PM), which offers a more inclusive alternative to the term ‘citizen science’. The Society for Participatory Medicine defines PM as:

*‘where patients and health professionals actively collaborate and encourage one another as full partners inhealthcare’*.^14^

For our purposes, we extended the term's meaning to denote inclusive, structured, and bi-directional collaboration across three key actors: lay contributors; clinicians/researchers; and intelligent systems. In doing so, we build on existing definitions of CS. Bonney, writing from ornithology, defined CS as a structured, collaborative process between scientists and the public, typically with an educational component,^3^ whereas Irwin, from a policy perspective, emphasised citizens’ roles in shaping science policy and the value of local and contextual knowledge.^15^ This distinction remains relevant in medicine, where systemic gender- and minority-based biases influence whose knowledge counts; for example, misdiagnosis of liver disease symptoms in women can occur because of male-normed clinical expectations.^16^ Our definition of PM draws from both perspectives while also incorporating intelligent systems as epistemic partners. The depth and structure of public involvement can vary greatly. For reference, we include the widely cited typology developed by Shirk *et al.*^8^ (Fig. 1A).

Rather than replacing traditional research, PM complements it, extending public participation across the research cycle and embedding intelligent systems as tools as well as facilitators and co-learners. Hepatology presents an ideal testing ground: liver diseases are widespread and often preventable,^17^ yet remain poorly understood by the public,^18^ underfunded and limited in available data. These challenges are precisely where PM can make a difference: by raising awareness, strengthening prevention, and engaging diverse audiences in shaping the future of hepatology. Building on the foundation laid by patient organisations, such as the European Liver Patients’ Association (ELPA), which have shown how collective advocacy can influence research and policy,^19^ PM widens participation to include not only patients, but also undiagnosed individuals, at-risk groups, and other lay contributors who bring new perspectives to liver research.

### The human-AI loop

In medical contexts, AI encompasses statistical and computational approaches used to detect patterns, classify signals, and make predictions across large datasets. In hepatology, these approaches are increasingly used for imaging, risk prediction, and text analysis.^20^ More recently, AI has transitioned from a one-way human-supporting tool to a co-learning partner, with humans and intelligent systems interacting in dynamic feedback loops.^21^ In research, AI increasingly functions within human-machine learning cycles, where humans train AI and AI, in turn, structures and supports human learning.^10^ Drawing on the *Zone of Proximal Development*, such co-learning frameworks assign tasks that stretch but do not overwhelm either learner. Gravity Spy^10^ illustrates this logic. The machine learning (ML) model routes high-confidence ‘glitches’ (noise artefacts in gravitational wave data) to beginners and more ambiguous cases to experienced volunteers, allowing newcomers to contribute quickly while improving performance. For instance, scaffolded volunteers achieved 95% accuracy (60% in controls), submitted nearly twice as many classifications (228 *vs.* 121) and corrected ML errors and flagged unfamiliar glitch types, which were used to expand the training set of the model. In effect, AI accelerates human learning by managing task difficulty, while humans extend the capacity of AI by supplying insight and novelty. A similar approach could support hepatology, where AI routes simple pattern-recognition tasks to non-specialists, whereas unusual cases are escalated to clinicians. Identifying such sweet spots of shared learning is key to future PM-AI partnerships.

### Motivation for participatory hepatology

#### Increasing awareness

Although public awareness of liver disease has increased modestly in recent years,^22^ it remains very low. Analyses of NHANES data show minimal gains. Singh *et al.*^23^ reported increases from 1.5% to 3.1% overall and from 1.6% to 3.4% among affected individuals (2001–2016; ∼7,000 participants). Alqahtani *et al.*^24^ reported similar limited awareness (4.4% to 6.3%), despite nearly 37% meeting metabolic dysfunction-associated steatotic liver disease (MASLD) diagnostic criteria^25^(2007–2016; ∼11,700 participants). Awareness was 0% among 18–29-year-olds, highlighting a knowledge deficit even in younger adults. Recent, smaller sample surveys show similar trends (*e.g.* Ajaz *et al.*^26^) Globally, awareness remains insufficient.^27^ In response, the EASL-Lancet Commission named education and awareness as top priorities for protecting future generations.^22^^,^^28^ Public engagement, particularly through participatory hepatology, offers a promising means of addressing this gap.

#### Increasing prevention

**Fig. 2:**
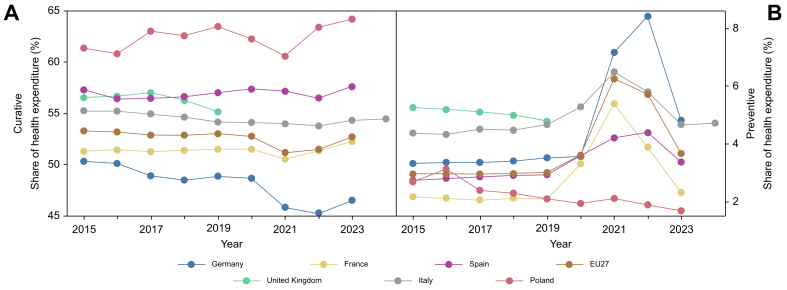
Share of health expenditures on curative and preventive treatments. Eurostat^30^ calculations regarding the share of the healthcare budget spent on curative (A) and preventive healthcare (B) for the five largest countries in the EU, the UK and the EU 27 average.

The lack of awareness is compounded by underinvestment in disease prevention, which receives limited political and societal support despite being life-saving and cost-effective.^29^ Across European Union (EU) member states, prevention is consistently underfunded.^28^^,^^30^ In the EU-27, ∼52-54% of health expenditure is allocated to curative care, whereas only ∼3-4% goes to primary prevention (aside from a brief COVID-19 spike^30^) (Fig. 2). Current data suggest that this increase was temporary and largely driven by testing and vaccination. The 2024 preventive health expenditure in Italy, for instance, appears to have stabilised at pre-pandemic levels. In France, the 2022 prevention budget supports this interpretation, with only 6% of €12.7 billion going to information and education, with 2.5% allocated to nutritional health and health promotion, two subcategories that are highly relevant to liver disease prevention. Around 40% of the French prevention budget (€5.1 billion) was allocated to early detection and secondary prevention, but spending on non-COVID disease detection remained similar to pre-pandemic levels, at 3.4%.^31^

Liver disease exemplifies the consequences of this underinvestment given that nine out of 10 cases are preventable.^32^ In the UK, liver-related health information is fragmented across NHS platforms, unlike the centralised campaigns for cardiovascular disease, diabetes, or cancer,^33^ despite a 93% increase in premature liver-related deaths over two decades.^34^ However, this figure might reflect, in part, changes in death certification practices, because underlying liver conditions were historically underreported. Nevertheless, liver disease is now the only major cause of death with steadily rising rates since the 1970s, a fourfold increase, whereas deaths from heart disease and diabetes have declined.^32^ Hospitalisations have doubled in a decade, and mortality is four times higher in the most deprived areas.^35^ Similar patterns might be emerging elsewhere in Europe.^36^

#### Increasing data availability

Barriers to awareness and prevention are reinforced by gaps in the evidence base. Most medical research relies on clinical data (*e.g.* hospital records, trials) or population cohorts (*e.g.* UK Biobank),^37^ often comparing diseased and control groups to identify statistically significant differences.^38^ Although randomised controlled trials remain the gold standard for assessing interventions, clinical data over-represent severely ill patients,^39^ and population cohorts over-represent healthier, wealthier, and health-conscious individuals.^40^ Attrition further introduces bias, because later waves may differ systematically, as seen in the Dutch Pienter Biobank.^41^ These limitations are problematic for conditions such as MASLD, metabolic dysfunction-associated steatotic liver disease + alcohol-associated liver disease (MetALD) or alcohol-associated liver disease (ALD), which are closely tied to lifestyle and socio-economic status.^42^ Statistical techniques such as inverse probability weighting can reduce, but not eliminate, these biases.^43^

PM offers a valuable complement. Although based on non-probability sampling, PM can expand data availability for under-represented groups^44^ and improve the accuracy of statistical estimates.^45^ Beyond quantity, it can provide context-rich, continuous, and flexible data that can respond to emerging questions. Given that PM engages lay contributors directly, it foregrounds how, by whom, and under what circumstances data are generated, adding insight into both technical and social dimensions. Although not a panacea, it offers an alternative pathway for strengthening and diversifying biomedical datasets.^46^

### A collective route to better liver health

Preventive healthcare is shaped by societal and political contexts. In some settings, prevention can be viewed as paternalistic interference with individual freedoms,^47^ whereas public disengagement with science and the proliferation of misinformation further undermine support for preventive measures.^48^ These challenges can be reduced when there is sufficient public awareness and acceptance of the benefits of a programme, leading to a groundswell of support that governance structures must respond to. Involving the public more closely in knowledge generation, through PM initiatives, not only enhances scientific outcomes, but also counters the perception of prevention as state imposed. Thus, prevention efforts become ‘wisdom of the crowd’ solutions shaped collaboratively by scientists and the public.

Here, we aim to advance PM in hepatology, focussing on its synergy with AI. We briefly present four exemplary case studies from astrophysics, molecular biology, meteorology, and traffic management. These case studies inspired four new public-led PM projects, *Liver Zoo,* *Liver Cache*, *LiverQuest*, and *Heporama*, and two additional patient-centric projects (*Liver Cancer Wisdom Bank* and *Liver4Mind*), as discussed below.

## Materials and methods

### Cross-disciplinary inspirations for liver health

We selected four CS^1^ projects that illustrate the breadth of possibilities for public participation in science and that could serve as inspiration for future hepatology-focused initiatives. To stimulate discussion of innovative approaches in hepatology, we drew on disciplines outside medicine, where CS traditions with respect to general public participation are already well established. Each project exemplifies distinct approaches to engaging the public. Although the tools through which citizens now participate in science have become ever more digital, ranging from online image classification to AI-enabled sensors, the underlying logic is unchanged: distributed perceptual intelligence, creative problem-solving, and the aggregation of many small contributions into shared evidence.

**Fig. 3:**
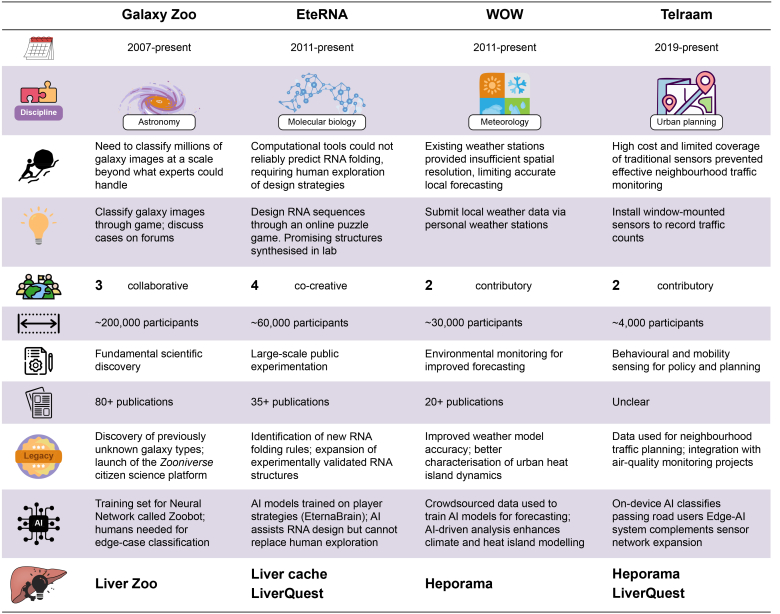
Overview of four citizen science projects outside medicine: *Galaxy Zoo*,^4^^,^^51^^,^^52^*EteRNA*^2^^,^^53^^,^^54^^,^^55^, *WOW*,^49^^,^[56], [57], [58] and *Telraam*.^50^^,^^59^^,^^60^ This summarises the key features, including time span, disciplinary focus, research goals, main challenges and solutions, project model,^8^ country of origin, participant numbers, outputs (publications, discoveries, or policy impact), and relevance for AI. In the last row, we specify for which hepatology projects they provided inspiration. AI, artificial intelligence.

Together, *Galaxy Zoo*,^4^ *EteRNA*,^2^ *WOW*,^49^ and *Telraam*^50^ demonstrate the multipronged nature of CS, each embodying a different mode of participation. These initiatives span advancing fundamental scientific discovery (*Galaxy Zoo*, involving volunteers classifying galaxies), enabling large-scale public experimentation (*EteRNA*, a gamified platform where players solve RNA-folding puzzles) and generating citizen-produced environmental and behavioural data aimed at informing policy and planning (*WOW*, integrating data from citizen-hosted weather stations and *Telraam*, where citizens deploy street-level traffic sensors). They illustrate the diverse aims that CS projects can serve. Their key features, such as scale and participation levels, are summarised in Fig. 3. These projects will serve as conceptual anchors for four of the six creative projects we propose in hepatology, thereby offering inspiration for other researchers (see Results Section). The approaches range from gamified experimentation (*LiverQuest* and *Liver Cache*) to citizen-generated environmental and behavioural data (*Heporama* and *Liver Cache*) and distributed perceptual intelligence (*Liver Zoo*).

### Design criteria for hepatology projects

While the preceding subsection focused on cross-disciplinary sources of inspiration and their modes of public participation, here we describe the principles used to delimit the set of PM projects. We designed six PM projects that span the main types of participatory initiative relevant to hepatology. Each addresses a different dimension of liver-disease risk: behavioural and lifestyle factors (*LiverQuest*); perceptual and phenotypic pattern recognition (*Liver Zoo*); environmental and exposure-related risks (*Heporama*); community-level and structural influences (*Liver Cache*); lived-experience and patient-reported insights (*Liver Cancer Wisdom Bank*); and psychosocial, cognitive, and stigma-related determinants (*Liver4Mind*). The six projects also map onto different points along the liver-disease trajectory, spanning upstream determinants (behavioural, environmental, and psychosocial), midstream opportunities for early detection (visual or phenotypic cues), and downstream challenges linked to cancer risk, lived experience, and care pathways.

Selection followed four criteria, which were intended to act as conceptual guardrails, ensuring relevance, feasibility and ethical plausibility rather than readiness for immediate deployment: (1) each project targets a documented gap in liver-health prevention, surveillance, or patient support where public participation provides added value; (2) each proposal adapts mechanisms proven effective in existing CS projects (*e.g.* *Galaxy Zoo*, *EteRNA*, *WOW*, and *Telraam*), ensuring conceptual and technical plausibility; (3) the set of projects spans increasing implementation complexity, from low-cost behavioural and annotation tasks to more demanding sensing and modelling pipelines, so that near-term prototypes sit alongside more ambitious, ‘think-big’ ideas; and (4) the six projects collectively map onto major elements of liver pathology and its determinants, illustrating how PM can support primary and secondary prevention, environmental risk assessment, and patient centred insights across the disease course.

## Results

### Participatory sensing, imaging, and gamification for liver health

#### Liver Zoo

**Rethinking visual attention in liver imaging.** Medical imaging is central to the assessment of liver disease, particularly through ultrasound, but also magnetic resonance and computer tomography.^61^ Image interpretation is typically performed by radiologists or increasingly by AI systems. Both rely on systematic approaches: structured reporting improves clarity, ensures clinically important features are consistently addressed, enhances communication with referring clinicians, and supports AI-driven workflows.^62^ These advantages explain why structured templates are adopted across radiology. At the same time, predefined structures, whether in reporting templates or in AI models, necessarily emphasise recognised diagnostic targets. This focus can make subtle, ambiguous, or unexpected patterns less likely to be highlighted. Likewise, deep learning models perform well on predefined tasks, with diagnostic Areas Under the Curve (AUC) often exceeding 80–95%^63^ on targeted tasks. These are normally optimised for specific tasks and, hence, exclude features outside their scope. Consequently, subtle or ambiguous features, such as faint peripheral banding, asymmetric texture, or early signal dropout, might be visible yet are rarely modelled.^64^ However, these imaging signals can correspond to early indicators of disease or even novel subtypes.

***Galaxy Zoo*** **as an informative precedent.** Inspiration for *Liver Zoo* came, in part, from *Galaxy Zoo*. Some of its most important results arose when volunteers began to notice strange objects that did not fit the provided categories. The informal observations, which were discussed in project forums, led to the identification of entirely new classes of astronomical phenomena.^51^ These discoveries showed that distributed, collective observation by people who were free to pay attention to what seemed strange, could achieve important results. In other words: discovery also happened by allowing people to look beyond what they were told to find.

**Fig. 4:**
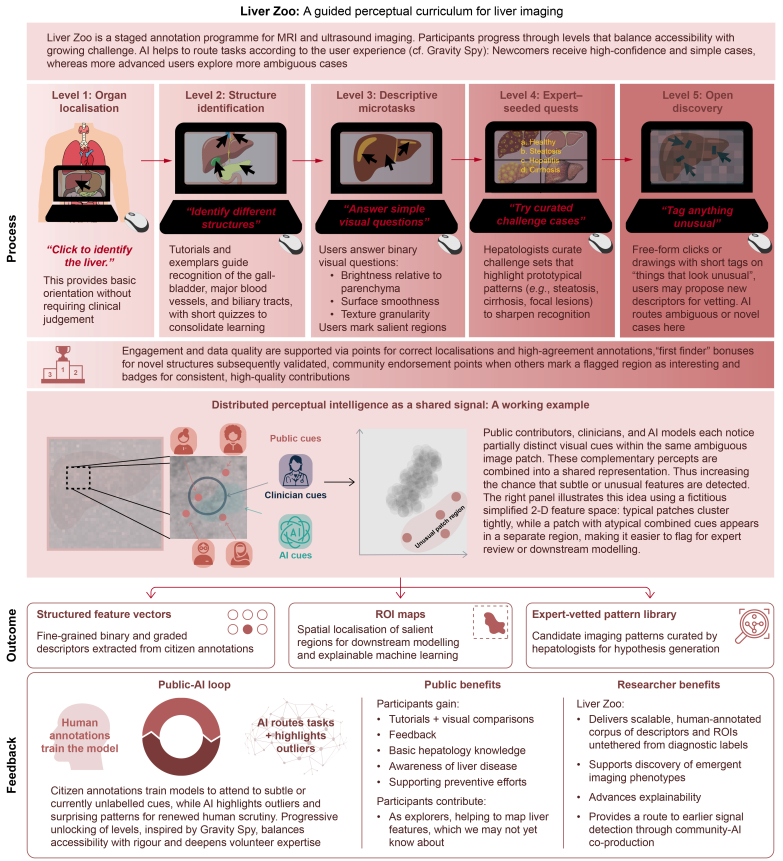
Schematic overview of *Liver Zoo*. This figure displays the process, outcome, and feedback loops, demonstrating what such a PM project could look like. Additional references include.^10^ AI, artificial intelligence; PM, participatory medicine.

The idea behind *Liver Zoo* is to complement expert review and diagnostic AI by opening new areas of inquiry. All images used in such a project would be fully anonymised, ensuring that privacy and confidentiality are protected. Over time, aggregated citizen inputs (*e.g.* simple annotations of brightness, texture, or unusual shapes) could help generate hypotheses about early phenotypes, produce datasets for training explainable AI, or reveal imaging signals linked to disease trajectories. This approach fits within the broader framework of PM by allowing laypeople to perceptually explore rather than diagnosing. This approach also supports a dynamic loop between human and machine: public inputs train AI to detect overlooked features, whereas AI highlights surprising patterns for renewed human scrutiny. Hence, *Liver Zoo* broadens both the scope of liver imaging science and the community engaged in it (Fig. 4).

#### Liver Cache

Medical students often learn about liver disease in clinical settings, far removed from the environments that shape patients’ lives. Yet, liver health is also influenced by social, environmental, and emotional contexts ranging from stigma and diet to housing and urban neglect.^65^ This disconnect can limit students’ understanding of disease as more than just a biological process. The second reason for devising this project can be viewed entirely within the context of some of the challenges set out in the Introduction, such as low awareness, prevention, and data availability. Therefore, we do not reiterate this knowledge gap here, but refer the reader back to the context described in the aforementioned section.

**Fig. 5:**
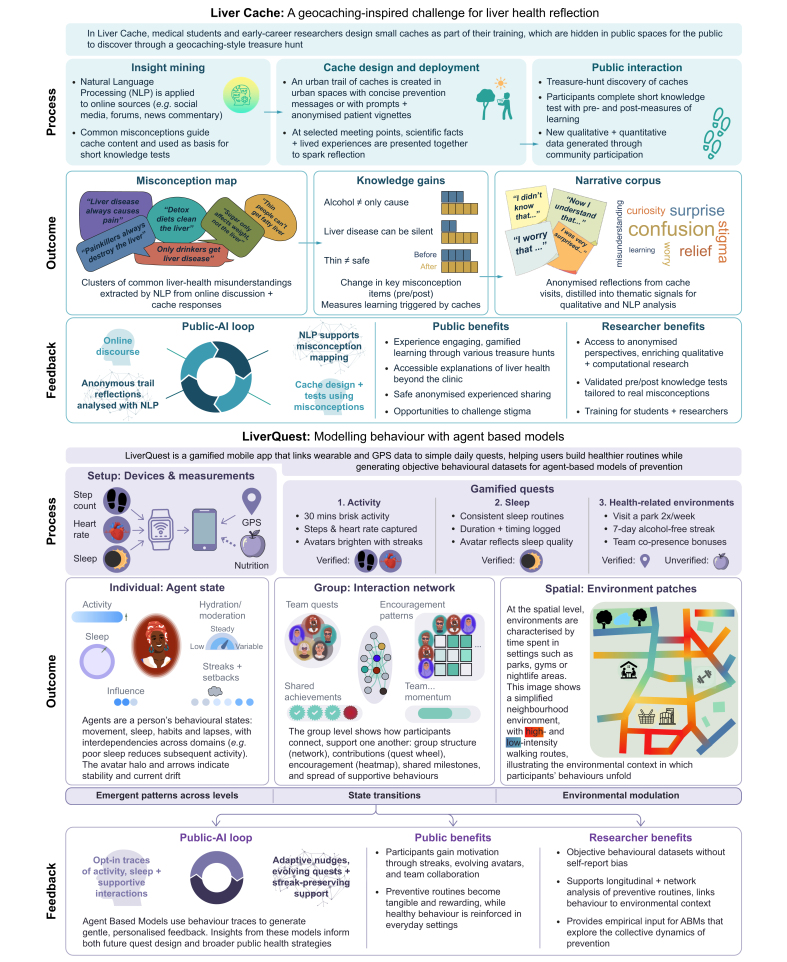
Schematic overview of *Liver Cache* and *LiverQuest*. This figure displays the process, outcome, and feedback loops, demonstrating what such a PM project could look like. Additional references include.^69^ AI, artificial intelligence; PM, participatory medicine.

One way to address both the educational gap and limited public awareness is to draw on gamification, which has been shown to enhance public engagement and awareness in health and science communication.^66^ Within this field, geocaching, a GPS-guided treasure hunt, has already been applied to public health, for instance to raise awareness of automated external defibrillators (AED) through caches in Ireland, UK, Czechia, and the USA^67^ and to engage the public with climate change through location-based challenges.^68^ Building on these examples, *Liver Cache* adapts geocaching to liver health, turning the city into a discovery site as well as a place where reflection can take place, not only for the public, but also for medical students who design and maintain the caches as part of their training (Fig. 5).

#### LiverQuest

**Limits of** **self-reported** **data.** A persistent challenge in prevention research is the reliance on self-reported behaviour, which is prone to recall bias, underestimation, and selective reporting. These shortcomings can undermine validity of findings and make it difficult to model long-term behavioural dynamics. Examples include dietary intake questionnaires that often underestimate caloric and alcohol consumption^70^ and intensity, or self-reported sleep diaries that diverge substantially from objective measurements.^71^ By contrast, wearables and GPS tracking provide objective measures of activity, sleep, and location. Studies in environmental health and physical activity research have shown that such data can reliably classify visits to health-relevant environments, such as parks, gyms, workplaces, and nightlife districts.^69^ Besides strengthening validity, such data streams open the door to behavioural simulation approaches that move beyond individual self-reports (Fig. 5).

**Agent-based modelling interventions.** Agent-based models (ABMs) provide a methodological framework for analysing such data. Unlike predictive AI, which focusses on estimating individual risk, in ABMs, individuals are represented as ‘agents’ with behavioural rules (*e.g.* how often they exercise, how they respond to peers, or how they relapse after stress).^72^ These agents interact with each other and with their environments, producing complex, large-scale, collective patterns. Such models have long been used in epidemiology to study how contagion spreads from individual behaviour.^73^ However, a recurring limitation is that ABMs often rely on sparse data, which weakens their realism and predictive power. Acquiring detailed, longitudinal traces of daily routines, social interactions, and environmental context is difficult outside of specialised cohorts. In prevention research, this makes it challenging to capture how routines spread, how relapse clusters emerge, and how interventions might reshape trajectories at the population level. Objective data from wearables and GPS, as envisioned in *LiverQuest*, can help overcome this gap by grounding ABMs in real-world behavioural dynamics.

**Behavioural change through gamification.** Thus, the challenge is how to generate rich behavioural data at scale. Gamification offers one solution. The launch of Pokémon Go showed how location-based augmented reality play could reach a wide audience and alter daily behaviour. Millions walked further, changed routines, and explored unfamiliar areas, through curiosity and attachment to virtual companions.^74^ This briefly achieved what health campaigns often fail to (*i.e.* motivating less active individuals to adopt preventive habits). Other games highlight complementary principles, such as the language-learning app Duolingo, which demonstrates how small daily rewards sustain long-term practice, while Tamagotchi showed how simple companions create emotional attachment and responsibility. Together, these examples illustrate three elements relevant to gamification within prevention: immersion in daily life; reinforcement of small habits; and motivation through attachment.

**From games to structured science.** CS projects illustrate how such engagement can also generate structured scientific knowledge. *EteRNA* turned puzzle-solving into experimental advances in RNA biology by creating a loop between lay exploration and lab validation.^2^^,^^53^ Sustained play produced high-volume, interpretable data of genuine scientific value. Three of its design principles translate well to public health prevention. First, exploration-led learning allows players to acquire knowledge by interacting as opposed to being instructed. Second, high-volume and diverse data are produced because continued play generates scientifically usable data. Finally, engagement through community features maintains player motivation and social interaction. These principles directly inform the design of *LiverQuest* (Fig. 5).

#### Heporama

**Aflatoxins and hepatocellular** **carcinoma.** Globally, hepatocellular carcinoma (HCC) is the fifth most common malignancy and the third leading cause of cancer-related death,^75^ exceeded in mortality only by lung and colorectal cancer. Accounting for 80–90% of all liver cancer mortality, HCC caused ∼760,000 deaths in 2022.^75^ Around 80% of these deaths occurred in Africa and Asia,^76^ with liver cancer ranking as the second most common cause of cancer mortality in Asia.^75^ Many different external risk factors have been identified that raise the risk of developing HCC (for a recent review, see^77^). Amongst these, aflatoxin exposure has been documented as being highly carcinogenic. Prior meta-analyses have estimated that the risks resulting from exposure to aflatoxins exposure will rise by a factor of 6 (95% CI 4–10).^78^

Aflatoxins are produced by *Aspergillus* fungi in improperly dried or stored grains, such as maize or nuts.^77^ This is especially prevalent in regions that are hot and humid. Climate change is responsible for raising temperatures globally and producing more volatile weather, which is likely to exacerbate this existing problem.^77^ Despite the serious risks posed by aflatoxins, systematic, community-level surveillance remains sparse in many low- and middle-income regions, because of inadequate monitoring infrastructure, limited public health investment, and fragmented data collection systems.^79^

**Community forecasting for prevention.** Despite Asia bearing the greatest global burden of HCC,^80^ one idea is PM involvement in rural Africa. This is because of the documented success of community-based CS initiatives in rural communities and in public health-related domains. An extensive overview of ∼30 CS projects in Africa is available in the appendices of,^81^ which suggest that rural, infrastructure-constrained environments in Africa are particularly well suited to the type of participatory environmental monitoring we propose.

**Fig. 6:**
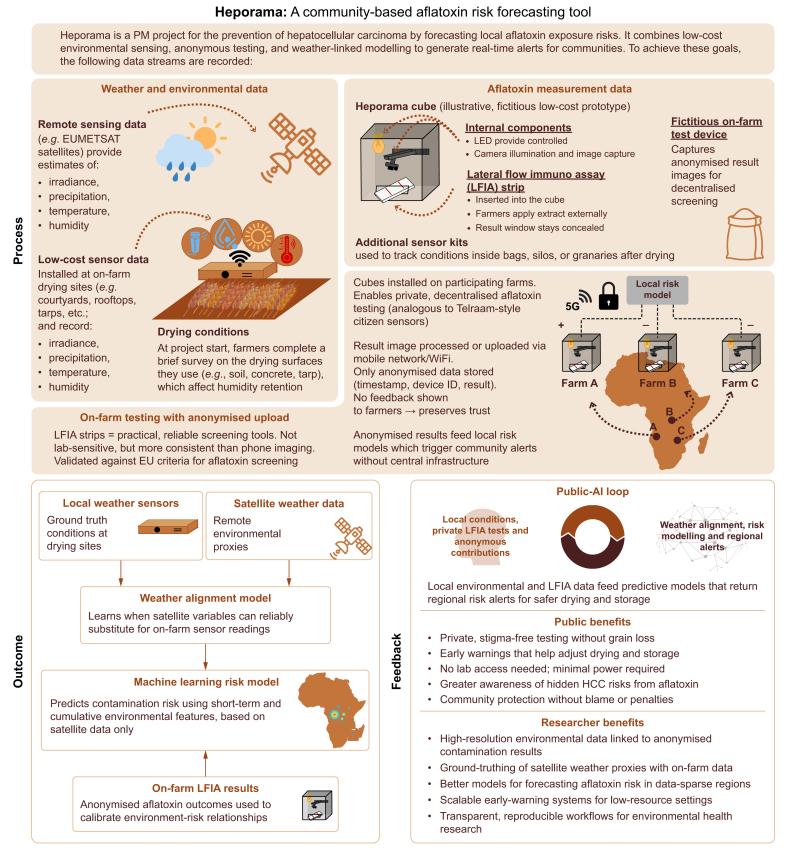
Schematic overview of the *Heporama* project. This figure displays the process, outcome, and feedback loops, demonstrating what such a PM project could look like. Additional references include.^49^^,^^50^^,^[82], [83], [84], [85], [86] AI, Artificial Intelligence; HCC, hepatocellular carcinoma; PM, participatory medicine.

Our proposed participatory aflatoxin risk forecasting project, which we call *Heporama*, draws inspiration from two proven CS projects in Europe, detailed above. As these examples illustrate (*i.e.* *Telraam* through community traffic sensing and *WOW* through citizen weather reporting), distributed monitoring can generate scientifically robust data that complement institutional models. *Heporama* adapts similar principles to the challenge of aflatoxin monitoring (Fig. 6).

### Empowering patients: New modes of participatory care

#### Liver Cancer Wisdom Bank

Most studies in hepatology focus on measurable variables, such as biomarkers, imaging, or prognostic scores, and qualitative research is often limited to small sample sizes.^87^ However, in liver cancer, patients face challenges that extend far beyond clinical parameters: fatigue, uncertainty, stigma, financial strain, and difficulties navigating complex health systems.^88^ These dimensions are rarely captured systematically.^89^

The *Liver Cancer Wisdom Bank* addresses this gap by collecting short voice recordings. Transcribed and structured reflections generate a searchable corpus of narratives, which can be linked to blood values and imaging data. This integration enables quantitative analysis of narrative features, correlation with cancer progression, and hypothesis-driven research on systemic barriers, psychosocial trajectories, and outcomes beyond survival.

**Fig. 7:**
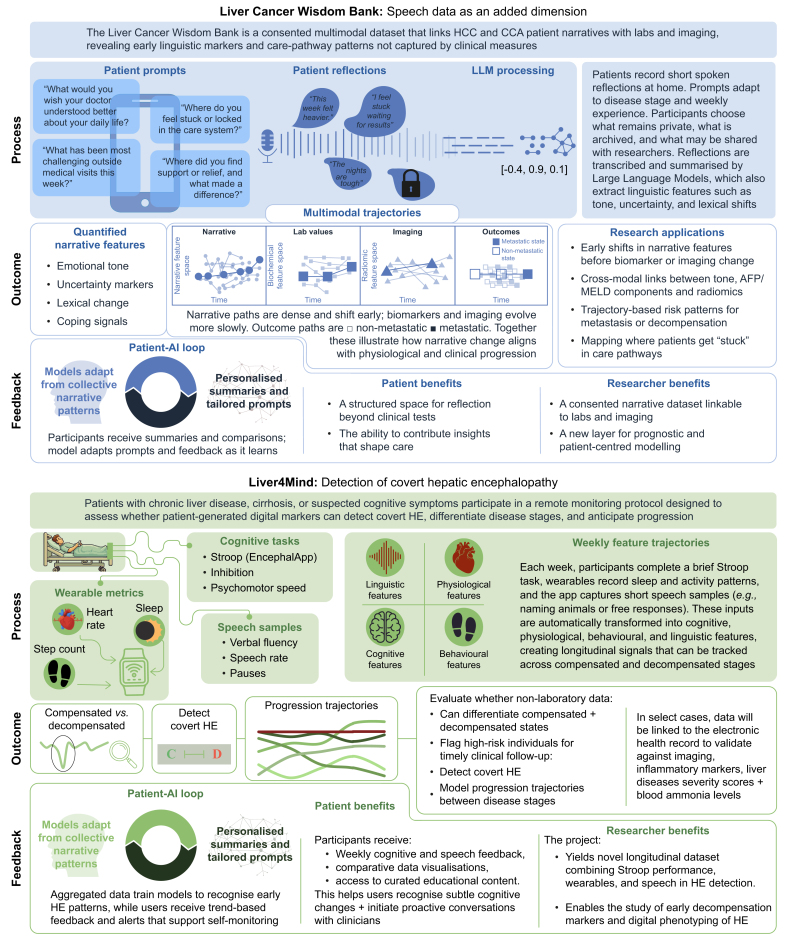
Schematic overview of the patient-centric PM projects: *Liver Cancer Wisdom Bank* and *Liver4Mind*. This figure displays the process, outcome and feedback loops, demonstrating what such a PM project could look like. AI, artificial intelligence; HCC, hepatocellular carcinoma; HE, hepatic encephalopathy; PM, participatory medicine; CCA, Cholangiocarcinoma.

Modules such as *Liver Voices* (curated narratives for professional or public use) and *Liver Connect* (peer matching based on shared experiences) extend the approach from individual reflection to collective learning. In this way, the *Liver Cancer Wisdom Bank* creates a novel source of patient-centred data with structured narratives that complement biomarkers (Fig. 7).

#### Liver4Mind

Many cases of cirrhosis today are only after decompensation occurs (*i.e.* when complications, such as ascites, bleeding, or overt hepatic encephalopathy (HE) make hospital contact necessary).^90^ Yet, all patients with decompensated cirrhosis have previously been in a compensated stage, a clinically silent period during which early intervention can improve prognosis and reduce healthcare utilisation.^91^

One of the few detectable complications of compensated cirrhosis is covert HE, a neuropsychiatric state marked by subtle deficits in attention, executive function, and psychomotor speed.^92^ These symptoms are often not evident without structured testing. Validated tools, such as the Stroop test counterbalanced by animal naming test to prevent learning effects, Psychometric Hepatic Encephalopathy Score (PHES) and Critical Flicker Frequency (CFF) can identify covert HE, but are rarely deployed outside specialty care. This leaves a major diagnostic blind spot: compensated cirrhosis is both the most treatable and the most underdiagnosed form of advanced liver disease.^93^ Thus, new approaches are urgently needed to identify these patients before decompensation occurs.

*Liver4Mind* proposes a novel, patient-led model for early detection and characterisation of compensated cirrhosis and covert HE. Unlike hospital-based monitoring platforms, it combines cognitive testing and wearable data in a longitudinal monitoring protocol that can be deployed in everyday life. This model is both scalable and empowering, enabling patients to track their own data while also contributing to scientific understanding of early-stage liver dysfunction outside the clinic (Fig. 7).

## Discussion

### Designing the future of liver health

This work is intentionally conceptual in nature and does not report empirical evaluations of implemented interventions. Instead, it aims to articulate design principles, risks, and boundary conditions for participatory medicine in hepatology, recognising that healthcare contexts introduce ethical, interpretative, and governance constraints that differ fundamentally from other CS domains. Although each project addresses a specific gap in hepatology, they also bring their own practical, ethical, and logistical challenges. Some of these are unique to the project design, whereas others recur across multiple project ideas. Therefore, we begin by outlining project-specific considerations before turning to the broader challenges that typify more than one project.

### Project-specific considerations

In addition to the broader challenges discussed below, each project faces specific considerations.

#### Liver Zoo

Lay observers might never identify consistently novel imaging features, given the limited visual appeal of liver magnetic resonance imaging. They do not have the same visual appeal as galaxy images. In addition, access to anonymised datasets is technically and ethically complex; there is also a risk that identified patterns reflect artefacts rather than biological signals. Nevertheless, Gravity Spy demonstrates that even domains with abstract or less visually engaging data can be made appealing through thoughtful task design, scaffolding, and community interaction.

#### Liver Cache

The placement and maintenance of caches are vulnerable to vandalism, weather, and practical hurdles, such as upkeep. AI analysis of public reflections could misinterpret cultural nuance or emotional tone.

#### LiverQuest

Although designed for ‘learning by playing’ rather than didactic messaging, the game, if not designed in a very thoughtful and careful manner, could risk trivialising serious health issues. The game is also dependent on smartphones and physical mobility might exclude some demographics. For this to be successful for researchers, contextual data (*i.e.* background characteristics of each player) would be beneficial and this could run into privacy legislation.

#### Heporama

Even low-cost sensor deployment and Cube distribution require initial investment and logistical coordination, and maintaining farmer trust depends on avoiding perceived penalties for positive results; geospatial outputs must be handled carefully to prevent stigmatising high-risk regions.

#### Liver Cancer Wisdom Bank

Voice data are sensitive and prone to misinterpretation without cultural or personal context, and participation might be limited by language, culture, and digital access.

#### Liver4Mind

Remote cognitive and wearable measures can be affected by unrelated comorbidities, producing false positives, and multimodal patient data must be ethically integrated while ensuring clinical interpretability.

### General challenges and limitations

The challenges outlined below can be understood as guardrails intended to prevent harm, misinterpretation, or inequitable participation as participatory hepatology initiatives move from concept to implementation.

#### User-centred design and perceived benefits

A general challenge concerns whether CS applications create clear value for intended users. Therefore, integrating user-centred, participatory design principles is essential to ensure that the needs, expected benefits, and potential usage barriers within target groups are adequately reflected in the design.^94^ User-perceived benefits, such as competence, recognition, meaningful contribution, or social connectedness, should be explicitly incorporated where applicable, because they can provide additional motivation for participation and, thus, complement public-health benefits.^95^

#### Recruitment and sustained engagement

Projects such as *Liver Zoo*, *Liver Cache*, *LiverQuest*, and *Heporama* all depend on recruiting large and diverse participant pools and maintaining their engagement over time. Motivation might wane without continuous content renewal, gamification, or visible impact. Participation might skew toward already-engaged or lower-risk groups, leaving high-risk or marginalised communities under-represented and, therefore, defeating one of the purposes of the projects to start with. Thus, developing an engagement plan that outlines how different target groups are reached, supported, and motivated, and how engagement is monitored and adapted over time, is essential to mitigate these risks and ensure that participatory aims are met.

#### Data quality, validation, bias, and interpretability

*Liver Zoo* and *Heporama* face risks of bias or model drift if conditions change or data quality declines. Across projects, volunteer-generated data require careful validation to avoid artefacts or overinterpretation. Finally, the question of representativeness should be carefully addressed. Participation is often self-selected, and selection bias can limit generalisability. In projects that feed into predictive models, such bias can distort model performance.

#### Ethics, privacy, and consent

All projects raise privacy considerations: imaging data (*Liver Zoo*), public reflections (*Liver Cache*), movement/location tracking (*LiverQuest*), geospatial risk maps (*Heporama*), voice recordings (*Liver Cancer Wisdom Bank*), and multimodal patient data (*Liver4Mind*). Safeguarding participants while enabling useful analysis demands robust consent processes, anonymisation strategies, and clear communication about data usage.

#### Access and inclusivity

Device requirements and digital literacy barriers can limit participation in *Liver Cache*, *LiverQuest*, *Liver Cancer Wisdom Bank*, and *Liver4Mind*. Physical mobility constraints can further restrict participation in location-based projects. Cultural and language diversity can also affect expression and interpretation, particularly in narrative-focused projects.

#### Integration into existing systems

Institutional uptake might be slow. The annotations of *Liver Zoo* might not fit current clinical workflows, Environmental data from *Heporama* are likely to require cross-sector coordination. In addition, insights from the *Liver Cancer Wisdom Bank* might take time to incorporate into care models, whereas the multimodel outputs of *Liver4Mind* must be clinically interpretable and ethically integrated.

Taken together, these considerations highlight the importance of staged development and cautious implementation of participatory hepatology initiatives. Consistent with this staged approach, one of the proposed projects, *Liver Cache*, is being piloted in the city of Aachen, Germany, having recently obtained funding for its implementation. Therefore, we do not report empirical findings from this work here, but its inclusion underscores the feasibility of progressing from conceptual design to pilot implementation within the proposed framework.

## Conclusion: Toward participatory hepatology

**Fig. 8:**
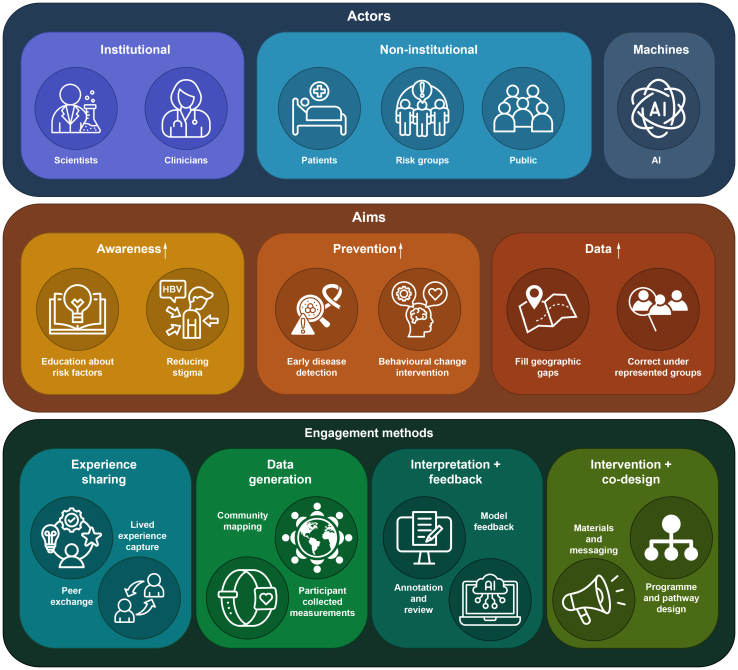
Our envisaged expanded PM framework. This figure shows the three main components of a PM framework with different forms of actors, aims, and engagement methods. AI, artificial intelligence; PM, participatory medicine.

In the Introduction, we set out three interconnected challenges in liver health: low public awareness; weak prevention uptake; and gaps in high-quality, representative data. Our six conceptual proposals address these challenges from multiple angles: public engagement; environmental and behavioural monitoring; and patient-led knowledge creation, while framing them within the broader concept of PM. This is summarised in Fig. 8 and can be used by the reader as an initial tool for devising a PM project in hepatology. By adapting proven participation models from other disciplines, these projects offer hepatology a practical path to bridge the gap between scientific research and the communities most affected by liver disease.

Liver disease is widespread, largely preventable, and offers important opportunities for prioritisation in participatory and public health frameworks. One way forward is to expand the role of the public and patients in shaping liver research, using models of participation adapted from other fields. We introduced an expanded framework of PM that brings scientists, laypeople, and AI systems together as active collaborators. Drawing from existing CS projects in astronomy, molecular biology, meteorology, and mobility, we developed six proposals for how similar forms of collaboration could be applied to hepatology: *Liver Zoo*, *Liver Cache*, *LiverQuest*, *Heporama*, *Liver Cancer Wisdom Bank* and *Liver4Mind*. These projects address a range of unmet needs: from raising awareness and reducing stigma, to environmental and behavioural data collection, to capturing patient insights and lived experience.

These proposals are not final solutions but working examples of what participatory research in hepatology might look like. Implementation will depend on ethical, logistical, and institutional factors, but the underlying message is simple: the separation between hepatology and the people most affected by it is not inevitable. Opening space for different kinds of questions, participants, and ways of knowing could strengthen both science and care. We warmly invite others in hepatology, public health, and patient advocacy to contact the authors and adapt or expand these and other ideas. Patients and the public are not outsiders to liver research because they are part of its future.

## Abbreviations

ABM, agent-based model; AED, automated external defibrillator; AI, artificial intelligence; ALD, alcohol-associated liver disease; CFF, Critical Flicker Frequency; CS, citizen science; ELPA, European Liver Patients’ Association; HCC, hepatocellular carcinoma; HE, hepatic encephalopathy; MASLD, metabolic dysfunction-associated steatotic liver disease; MetALD, metabolic dysfunction-associated steatotic liver disease + alcohol-associated liver disease; ML, machine learning; NAFLD, non-alcoholic fatty liver disease; PHES, Psychometric Hepatic Encephalopathy Score; PM, participatory medicine.

## Data availability statement

No new data were generated or analysed in support of this research.

## Authors’ contributions

Conceptualisation: BPML, CM, FPP, CVS. Writing - original draft: BPML, FPP, KA, CVS. Writing - review and editing: all authors.

## Declaration of generative AI and AI-assisted technologies in the writing process

During the preparation of this work, the author(s) used ChatGPT4o to improve the language and readability of the manuscript. After using this tool/service, the authors reviewed and edited the content as needed and take full responsibility for the content of the publication.

## Conflicts of interest

The authors declare no conflicts of interest.

Please refer to the accompanying ICMJE disclosure forms for further details.

## Supplementary data

Multimedia compoent 1

Multimedia compoent 2

## Footnotes

1 “Citizen science” is retained here for consistency with past descriptions; for our hepatology work we use PM.
