# Supplementary material for: The future of hepatology: Preventing liver disease together with the public, scientists and artificial intelligence
Source: JHEP Rep. 2026 Apr 22;8(8):101846. doi: 10.1016/j.jhepr.2026.101846 (PMC13380113; doi:10.1016/j.jhepr.2026.101846)
Supplement: Multimedia compoent 1 [file mmc1.pdf]

JHEP|Reports

JHEP Reports

CTAT methods

Tables for a "Complete, Transparent, Accurate and Timely account" (CTAT) are now mandatory for all revised submissions. The aim is to enhance the reproducibility of methods.

- Only include the parts relevant to your study
- Refer to the CTAT in the main text as 'Supplementary CTAT Table'
- Do not add subheadings
- Add as many rows as needed to include all information
- Only include one item per row

If the CTAT form is not relevant to your study, please outline the reasons why:

This manuscript is a conceptual paper focused on the design of new participatory medicine initiatives in hepatology. As such, it does not involve the use of any of the items outlined in 1.1-1.9. Neither is this paper concerned with Randomised Controlled Trials. Thus, none of these categories are applicable. Transparency and reproducibility are ensured through explicit description of concepts, sources, definitions, and methodological reasoning in the main text.

1.1 Antibodies

Name	Citation	Supplier	Cat no.	Clone no.

1.2 Cell lines

Name	Citation	Supplier	Cat no.	Passage no.	Authentication test method

1.3 Organisms

Name	Citation	Supplier	Strain	Sex	Age	Overall n number

1.4 Sequence based reagents

Name	Sequence	Supplier

1.5 Biological samples

Description	Source	Identifier

1.6 Deposited data

Name of repository	Identifier	Link

1.7 Software

Software name	Manufacturer	Version

1.8 Other (*e.g*. drugs, proteins, vectors etc.)

1.9 Please provide the details of the corresponding methods author for the manuscript:

2.0 Please confirm for randomised controlled trials all versions of the clinical protocol are included in the submission. These will be published online as supplementary information.

